# Altered microRNA expression profile in amyotrophic lateral sclerosis: a role in the regulation of NFL mRNA levels

**DOI:** 10.1186/1756-6606-6-26

**Published:** 2013-05-24

**Authors:** Danae Campos-Melo, Cristian A Droppelmann, Zhongping He, Kathryn Volkening, Michael J Strong

**Affiliations:** 1 Molecular Brain Research Group, Robarts Research Institute, University of Western Ontario, London, ON, Canada; 2 Department of Pathology, University of Western Ontario, London, ON, Canada; 3 Department of Clinical Neurological Sciences, Schulich School of Medicine and Dentistry, University of Western Ontario, London, ON, Canada; 4 University Hospital, LHSC, Rm C7-120, 339 Windermere Road, London, ON, N6A 5A5, Canada

**Keywords:** miRNA, Amyotrophic lateral sclerosis, Neurofilament

## Abstract

**Background:**

Amyotrophic Lateral Sclerosis (ALS) is a progressive, adult onset, fatal neurodegenerative disease of motor neurons. There is emerging evidence that alterations in RNA metabolism may be critical in the pathogenesis of ALS. MicroRNAs (miRNAs) are small non-coding RNAs that are key determinants of mRNA stability. Considering that miRNAs are increasingly being recognized as having a role in a variety of neurodegenerative diseases, we decided to characterize the miRNA expression profile in spinal cord (SC) tissue in sporadic ALS (sALS) and controls. Furthermore, we performed functional analysis to identify a group of dysregulated miRNAs that could be responsible for the selective suppression of low molecular weight neurofilament (NFL) mRNA observed in ALS.

**Results:**

Using TaqMan arrays we analyzed 664 miRNAs and found that a large number of miRNAs are differentially expressed in ventral lumbar SC in sALS compared to controls. We observed that the majority of dysregulated miRNAs are down-regulated in sALS SC tissues. Ingenuity Pathway Analysis (IPA) showed that dysregulated miRNAs are linked with nervous system function and cell death. We used two prediction algorithms to develop a panel of miRNAs that have recognition elements within the human NFL mRNA 3′UTR, and then we performed functional analysis for these miRNAs. Our results demonstrate that three miRNAs that are dysregulated in sALS (miR-146a*, miR-524-5p and miR-582-3p) are capable of interacting with NFL mRNA 3′UTR in a manner that is consistent with the suppressed steady state mRNA levels observed in spinal motor neurons in ALS.

**Conclusions:**

The miRNA expression profile is broadly altered in the SC in sALS. Amongst these is a group of dysregulated miRNAs directly regulate the NFL mRNA 3′UTR, suggesting a role in the selective suppression of NFL mRNA in the ALS spinal motor neuron neurofilamentous aggregate formation.

## Background

Amyotrophic lateral sclerosis (ALS) is a progressive, age-dependent, fatal disorder in which motor neurons within the brain and spinal cord (SC) degenerate. While the majority of cases are sporadic (sALS), 5 – 10% are familial (fALS) and include genes mediating a seemingly diverse number of processes. These include mutations in genes encoding Cu/Zn superoxide dismutase 1 SOD1; [1]; angiogenin [2,3]; *TARDBP* encoding TAR DNA binding protein 43 kDa or TDP-43; [4-6] , FUS/TLS encoding a RNA processing protein fused in sarcoma/translocated in liposarcoma; [7,8] and an expanded hexanucleotide repeat in the non-coding region of *C9ORF72*[9,10]. The pathology and alterations in expression of RNA binding proteins such as mutant SOD1 (mtSOD1), TDP-43 and FUS/TLS in ALS, as well as evidence of mutations in these proteins being associated with altered RNA processing, has suggested that alterations in the metabolism of RNA may be critical to the pathogenesis of ALS [1,6,8,11].

In addition, several of the proteins associated with ALS have the capacity to regulate the stability of the low molecular weight neurofilament (NFL) mRNA [12], of which the steady state levels are selectively suppressed in sALS [13-15]. These include mtSOD1 which greatly destabilizes NFL mRNA [16] and TDP-43 that stabilizes NFL mRNA [17]. We have also recently described a novel RNA binding protein, RGNEF (Rho guanine nucleotide exchange factor) that acts as NFL mRNA destabilizing factor and forms protein aggregates in ALS [18]. While the net effect of the coordinated action of these RNA binding proteins on NFL mRNA stability *in vivo* remains to be clarified, it is of interest that both NFL mRNA and TDP-43 are differentially partitioned to degradative granules (P-bodies) in ALS affected lumbar spinal motor neurons [12]. This is in contrast to the physiological partitioning of TDP-43 to stress granules observed following axonal injury [19,20]. Both stress granules and P-bodies exist in a constant state of dynamic flux, mediated by the nature of associated microRNAs miRNAs; [21,22].

MiRNAs are highly-abundant, highly-conserved small endogenous non-coding RNAs that participate in mRNA stability and translational regulation by either promoting degradation of mRNA, or by blocking translation without degradation [23]. Accumulating evidence supports a key role for miRNAs in all aspects of neuronal development, function and plasticity [24-28]. While a role for altered miRNA expression in a variety of neurodegenerative diseases is increasingly being recognized [29-35], nothing is known with respect to the miRNA expression profile in spinal cord (SC) in ALS and the role of miRNAs on NFL mRNA stability.

Here we used the quantitative RT-PCR based array method (TaqMan array) to investigate the profile of miRNA expression in ALS SC. Our results show significant differences in the expression of a large group of miRNAs in ALS SC lysates compared to controls. Among them is a population of miRNAs that have miRNA recognition elements (MREs) within the human NFL mRNA 3′UTR which we confirmed using functional analysis. Our data suggest a potential role of miR-146a*, miR-524-5p and miR-582-3p in the selective decrease of NFL mRNA observed in ALS that could contribute to the etiology of neurofilamentous aggregates and the pathology of ALS.

## Results

### sALS spinal cord has a characteristic miRNA expression profile

To characterize the expression pattern of known human miRNAs in sALS SC, RT-qPCR was performed using the TaqMan Low Density Human MicroRNA assay kit as described in the methods below. This allowed for the analysis of a total of 664 miRNAs from 3 cases of controls and 5 cases of sALS.

We observed 4 miRNAs expressed exclusively in sALS but not in controls [miR-558 (p < 0.001); miR-16-2*, miR-146a* (p < 0.01); miR-508-5p (p < 0.05)] (Table 1). In addition, 7 miRNAs were expressed in controls and not in sALS [miR-624 (p < 0.001); miR-520e, miR-524-5p, miR-548a-5p, miR-606, miR-612, miR-647(p < 0.05)] (Table 2). From 245 miRNAs expressed in both sALS and controls but at statistically different levels, 6 were expressed in sALS at higher levels than controls [miR-373*, miR-551a (p < 0.01); miR-506, miR-518a-5p, miR-518e*, and miR-890 (p < 0.05)] (Table 3) and all the remaining (239) were down-regulated in the sALS SC tissue (Additional file 1: Table S1). Only 10 miRNAs of the potential 664 miRNAs had no expression in sALS or control lumbar SC (Additional file 1: Table S2). To validate these observations, we randomly selected miRNAs from the results of the TaqMan array and subjected these to RT-qPCR using SYBR Green. We confirmed the significant alteration of these miRNAs in the SC in sALS (data not shown).

**Table 1:** MiRNAs expressed in sALS but not in control spinal cord lysates

**miRNA**	**P-value**	**Log**_**10**_**RQ**
16-2*	0.006	2.931744
146a*	0.002	2.617606
508-5p	0.018	1.689412
558	<0.001	5.733327

**Table 2:** MiRNAs expressed in control but not in sALS spinal cord lysates

**miRNA**	**P-value**	**Log**_**10**_**RQ**
520e	0.034	−1.743558
524-5p	0.037	−1.856280
548a-5p	0.018	−1.989360
606	0.027	−1.949588
612	0.036	−6.305046
624	<0.001	−3.218662
647	0.013	−1.368500

**Table 3:** MiRNAs expressed in both sALS and controls but up-regulated in sALS

**miRNA**	**P-value**	**Log**_**10**_**RQ**
373*	0.004	3.646303
506	0.040	0.395549
518a-5p	0.031	1.425218
518e*	0.020	2.497707
551a	0.009	0.989927
890	0.032	1.273212

These results demonstrate that a significantly altered miRNA expression profile exists in the SC in sALS.

### Dysregulated miRNAs in sALS are linked with nervous system function and cell death

To identify networks and biological functions related to the target genes of miRNAs differentially expressed in sALS versus controls, we used Ingenuity Pathway Analysis (IPA). A group of 256 miRNAs were analyzed. From these, 10 miRNAs were up-regulated in the SC in sALS (4 miRNAs expressed only in sALS and 6 miRNAs expressed in both sALS and controls but at higher levels in sALS) and all the remaining were down-regulated (7 miRNAs expressed only in controls and 239 miRNAs expressed in both sALS and controls but at higher levels in controls). The most enriched associated networks and biological functions amongst targets of dysregulated miRNAs are shown in the Table 4. MiRNAs up-regulated in sALS are associated with nervous system development and function. In addition, we identified inflammatory response within the top biological functions of the predicted targets of these miRNAs. As well, within the top network and biological functions of predicted targets of down-regulated miRNAs in sALS we found cell death, cellular growth and proliferation, inflammatory disease and nervous system development and function (Table 4).

**Table 4:** Top networks and biological functions of miRNAs dysregulated in sALS

**Top network functions for predicted targets of miRNAs up-regulated in sALS**		
**Associated network functions**	**Score**	
Cellular development, nervous system development and function, tissue development	3	
Cellular assembly and organization, lipid metabolism, nucleic acid metabolism	2	
**Top biological functions for predicted targets of miRNAs up-regulated in sALS**		
	**P-value**	**No. of molecules**
**Diseases and disorders**		
Reproductive system disease	2.13E^-03^-2.13E^-03^	2
Inflammatory response	2.92E^-03^-2.92E^-03^	1
Cancer	6.81E^-03^-4.35E^-02^	3
Hematological disease	6.81E^-03^-6.81E^-03^	1
Immunological disease	6.81E^-03^-6.81E^-03^	1
**Molecular and cellular functions**		
Antigen presentation	2.92E^-03^-2.92E^-03^	1
Cell to cell signaling and interaction	2.92E^-03^-2.92E^-03^	1
**Physiological system development and function**		
Hematological system development and function	2.92E^-03^-2.92E^-03^	1
Immune cell trafficking	2.92E^-03^-2.92E^-03^	1
**Top network functions for predicted targets of miRNAs down-regulated in sALS**		
**Associated network functions**	**Score**	
Cancer, gastrointestinal disease, hepatic system disease	26	
Cancer, reproductive system disease, connective tissue disorders	24	
Cell death, cancer, cell cycle	18	
Cancer, reproductive system disease, gastrointestinal disease	18	
Reproductive system disease, cancer, cellular growth and proliferation	11	
**Top biological functions for predicted targets of miRNAs down-regulated in sALS**		
	**P-value**	**No. of molecules**
**Diseases and disorders**		
Reproductive system disease	4.31E^-88^-2.58E^-03^	93
Inflammatory disease	3.04E^-47^-4.99E^-02^	55
Renal and urological disease	3.04E^-47^-3.95E^-03^	52
Cancer	6.09E^-41^-4.40E^-02^	81
Gastrointestinal disease	1.44E^-21^-1.25E^-02^	48
**Molecular and cellular functions**		
Cellular development	1.51E^-07^-4.40E^-02^	22
Cellular growth and proliferation	1.51E^-07^-4.99E^-02^	31
Cell cycle	2.16E^-05^-4.40E^-02^	11
Cellular movement	5.09E^-04^-4.99E^-02^	15
Cell death	7.84E^-04^-4.10E^-02^	12
**Physiological system development and function**		
Organismal development	2.33E^-11^-6.61E^09^	7
Connective tissue development and function	2.16E^-05^-2.16E^-05^	4
Hair and skin development and function	8.96E^-03^-8.96E^-03^	1
Nervous system development and function	8.96E^-03^-8.96E^-03^	1
Respiratory system development and function	8.96E^-03^-3.54E^-02^	3

These results indicate that miRNAs dysregulated in sALS could potentially be involved in the biological functions affected in the ALS pathology.

### MiRNAs differentially expressed in sALS have MREs in the human NFL mRNA 3′UTR

We used two computational algorithms (microRNA.org, TargetScan) to develop a predicted panel of miRNAs that have differential expression in sALS and MREs within the human NFL mRNA 3′UTR (Table 5). This focus on NFL mRNA was driven by our previous observations of a selective suppression of NFL mRNA in spinal motor neurons in ALS [13-15]. For predicting MREs we considered the three NFL 3′UTRs reported: NFL 3′UTR-Short (−S; 286 b), NFL 3′UTR-Medium (−M; 1380 b) and NFL 3′UTR-Long (−L; 1838 b). We took into account only miRNAs with perfect pairing of their seed regions to their respective MREs within the NFL 3′UTR, and either conserved or not conserved MREs but always with good mirSVR scores and the most favorable Gibbs free energies (ΔGs) required for the interaction.

**Table 5:** MiRNAs differentially expressed in sALS and controls, and predicted recognition elements (MRE) in NFL 3′UTR

**miRNA**	**Expression in sALS compared to controls**	**NFL mRNA 3′UTR target**	**Conservation of MREs**
23a	down	S/M/L	Conserved
23b	down	S/M/L	Conserved
30a	down	S/M/L	Conserved
30b	down	S/M/L	Conserved
146a*	up	M/L	Not conserved
192	down	M/L	Conserved
193a-5p	down	M/L	Not conserved
215	down	M/L	Conserved
524-5p	down	L	Not conserved
556-5p	down	M/L	Not conserved
582-3p	down	S/M/L	Not conserved

**Figure 1:**
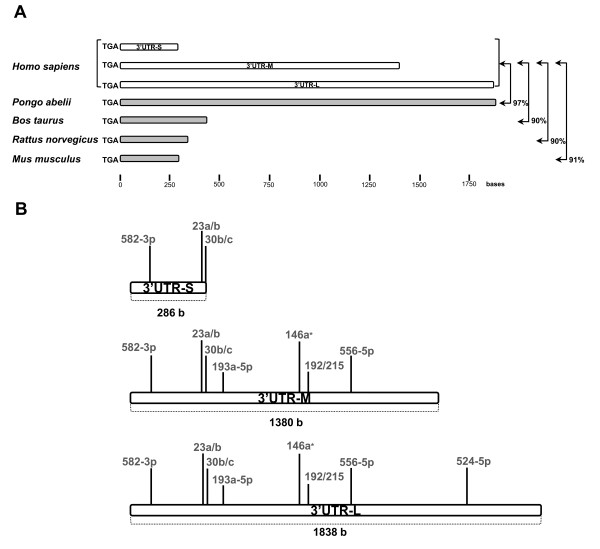
**Illustration of the sequence alignment of the NFL mRNA 3′UTRs of different species.** (**A**) Percentages of sequence identity between *Homo sapiens* and other mammals are indicated. (**B**) MREs within the different NFL mRNA 3^′^ UTRs. Note that not all miRNAs have MREs within the three NFL 3′UTRs reported (−S, -M and –L).

Using the above restrictions, the only miRNA that had an MRE in the NFL mRNA 3′UTR and was up-regulated in sALS was miR-146a*. All miRNAs down-regulated in sALS had only one recognition element within the NFL mRNA 3′UTR. Although the sequence identity between NFL mRNA 3′UTRs among *Homo sapiens* and other mammals is over 90% (Figure 1A), we found that some miRNA recognition sites are conserved but others are not (Table 5). It is important to note that some miRNAs have MREs within the three NFL 3′UTRs reported, -S, -M and –L (e.g. miR-23a), others within NFL 3′UTR-M and –L (e.g. miR-146a*) and one miRNA has a single recognition element within the NFL 3′UTR-L (miR-524-5p; Table 5, Figure 1B).

These results show that not only is the miRNA expression profile altered in ALS, but that miRNA that are predicted to target NFL mRNA 3′UTR are amongst those that are altered.

### MiRNAs differentially expressed in sALS regulate the expression of a reporter coupled to NFL mRNA 3′UTR

To study the functional relevance of those miRNA with altered expression in sALS on the regulation of the NFL mRNA 3′UTR, we used luciferase reporter gene assays and relative quantitative RT-PCR. We chose to examine all three lengths of 3′UTR to provide information about the availability of MREs to interact in the context of distinct mRNA structures. We transfected HEK293T cells with pre-miRNAs and a reporter vector harboring one of the three human NFL mRNA 3′UTR (−S, -M or -L).

In the reporter assay, we focused on two patterns: miRNAs up-regulated in sALS that induced down-regulation of the firefly luciferase activity, and miRNAs down-regulated in sALS that induced up-regulation of the luciferase activity. Both cases could explain the selective suppression of NFL mRNA observed in the SC in sALS.

**Figure 2:**
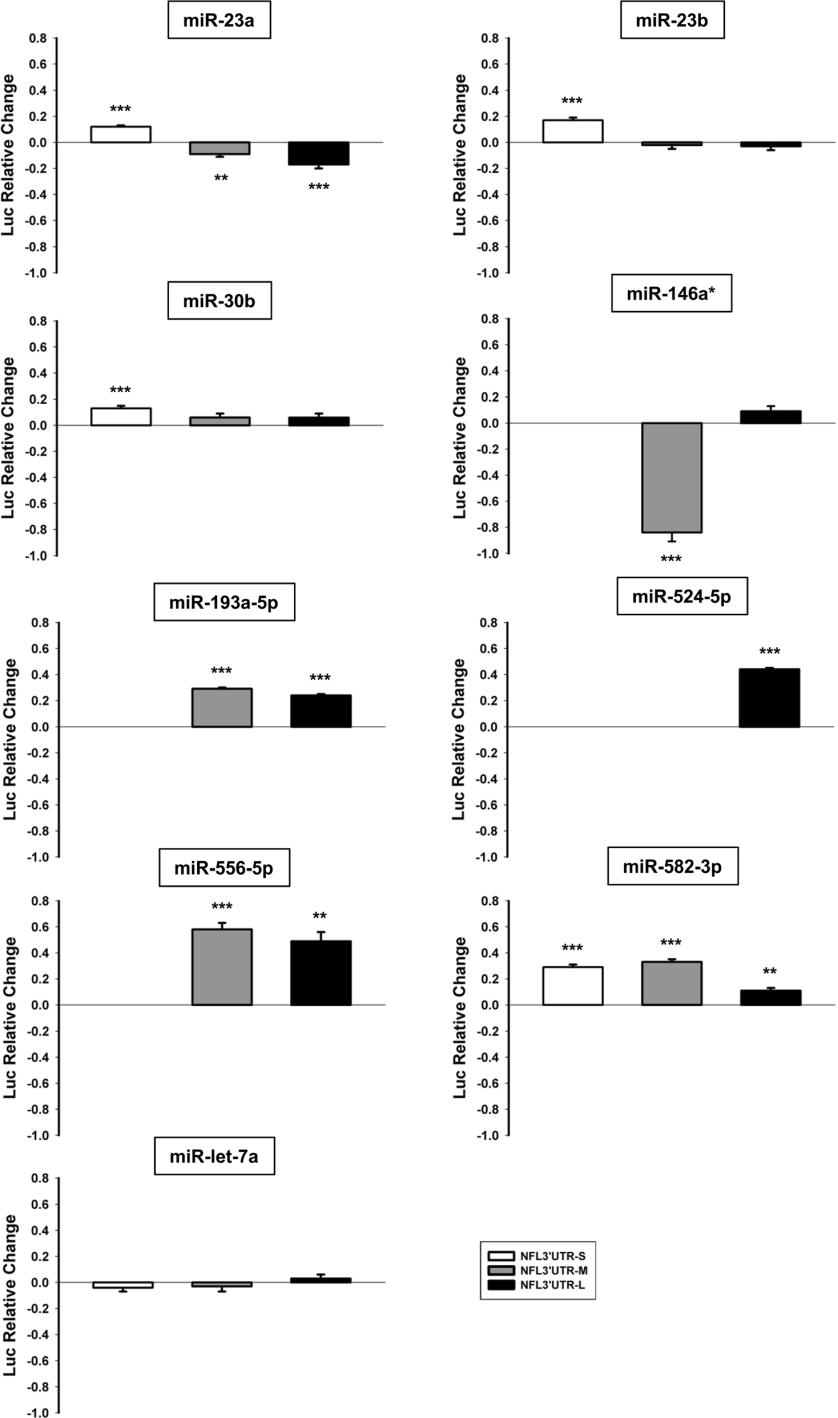
**A pool of miRNAs dysregulated in sALS regulates the activity of a reporter linked to NFL 3′UTR.** Reporter gene assay was performed transfecting HEK293T cells with pre-miRNAs and a reporter construct expressing firefly luciferase coupled to NFL 3′UTR-S (white bar), -M (grey bar) or -L (black bar). The results of firefly luciferase activity were normalized with Renilla reporter luciferase activity and with the effect of each miRNA on the luciferase mRNA alone (pmirGLO vector control). Data are expressed as relative change and show positive values as up-regulation and negative values as down-regulation. Experiments were performed in triplicate. Result are shown as mean ± SEM (*t*-test: *** = p < 0.001; ** = p < 0.01, relative to the pmirGLO vector control). Note that not all miRNA have predicted interaction sites in all lengths of NFL 3′UTR, only data for which a predicted site is present are shown. In addition, only miRNAs with correlation among the results of the reporter gene assay and the expression observed in sALS are presented.

Reporter gene assays showed that the activity of firefly luciferase was significantly decreased with miR-146a* (p < 0.001; up-regulated in sALS; Table 1) when the reporter gene was bound to NFL mRNA 3′UTR-M (Figure 2). Interestingly, this down-regulatory effect was not seen in the case of miR-146a* on the reporter containing the NFL 3′UTR-L. In contrast, miR-23a, miR-23b and miR-30b (down-regulated in sALS; Additional file 1: Table S1) induced an increase of the luciferase activity when the reporter was linked only to NFL 3′UTR-S (p < 0.001; Figure 2). In addition, miR-193a-5p and miR-556-5p were also down-regulated in sALS (Additional file 1: Table S1) and showed an up-regulatory effect on the NFL 3′UTR-M (p < 0.001) and -L (p < 0.001 and p < 0.01, respectively; Figure 2). Finally, miR-524-5p and miR-582-3p (down-regulated in sALS; Table 2 and Additional file 1: Table S1) induced an up-regulatory effect on the NFL 3′UTR-L (p < 0.001), and NFL 3′UTR-S (p < 0.001), -M (p < 0.001) and –L (p < 0.01; Figure 2), respectively. MiR-30c, miR-192 and miR-215 did not show correlation between the results of the reporter gene assay and the expression levels observed in sALS that could explain the suppression of NFL mRNA observed in sALS SC (data not shown). No relative change in the levels of the reporter linked to the NFL 3′UTR was found in cells transfected with miR-let-7a (negative control; Figure 2).

**Figure 3:**
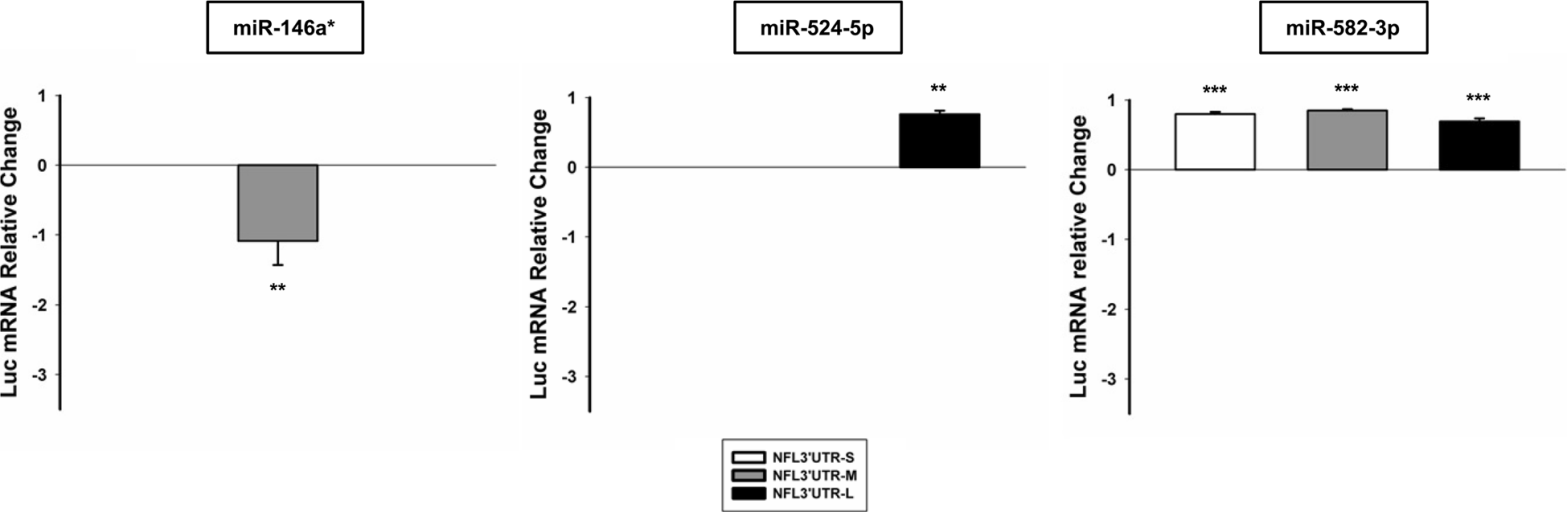
MiR-146a*, miR-524-5p and miR-582-3p regulate the expression of NFL mRNA 3′UTR. Relative quantitative RT-PCR was performed after the co-transfection of HEK293T cells with pre-miRNAs whose function was correlated with the differential expression observed sALS and a reporter construct expressing firefly luciferase coupled to NFL 3′UTR-S (white bar), -M (grey bar) or -L (black bar). Firefly luciferase was normalized to Renilla luciferase, the impact of each miRNA on the luciferase mRNA without NFL 3′UTR (pmirGLO vector control), and the effect of miR-let-7a (miRNA control) on the luciferase mRNA to obtain the specific effect on the NFL mRNA 3′UTR. Data are expressed as relative mRNA level change and show up-regulation as positive values and down-regulation as negative values. Experiments were performed in triplicate. Result are shown as mean ± SEM (*t*-test: *** = p < 0.001; ** = p < 0.01, relative to the pmirGLO vector control). Note that only results for those miRNAs that showed RT-PCR results in agreement with the reporter gene assay data are presented.

To elucidate whether the group of functional miRNAs were acting on NFL mRNA stability or translational regulation, we studied changes on the luciferase mRNA levels caused by NFL 3′UTR regulation through miRNAs. Relative quantitative RT-PCR showed that miR-146a* was able to decrease the level of firefly luciferase mRNA fused with NFL 3′UTR-M (p < 0.01; Figure 3), while miR-524-5p and 582-3p were able to increase the level of luciferase mRNA coupled to NFL 3′UTR-L (p < 0.01) and NFL 3′UTR-S (p < 0.001), -M (p < 0.001) and –L (p < 0.001), respectively (Figure 3). The remaining miRNAs are not presented because they did not show significant differences in the RT-PCR, or showed RT-PCR results in disagreement with the reporter gene assay that could be explained by an additional miRNA effect (direct or indirect) on translation (data not shown).

Therefore, miR-146a*, miR-524-5p and miR-582-3p showed consistent results in the reporter gene assay and the relative quantitative RT-PCR, making them prime candidates for the negative regulation of NFL mRNA expression in the SC in ALS.

### MiR-146a*, miR-524-5p and miR-582-3p directly regulate NFL expression

**Figure 4:**
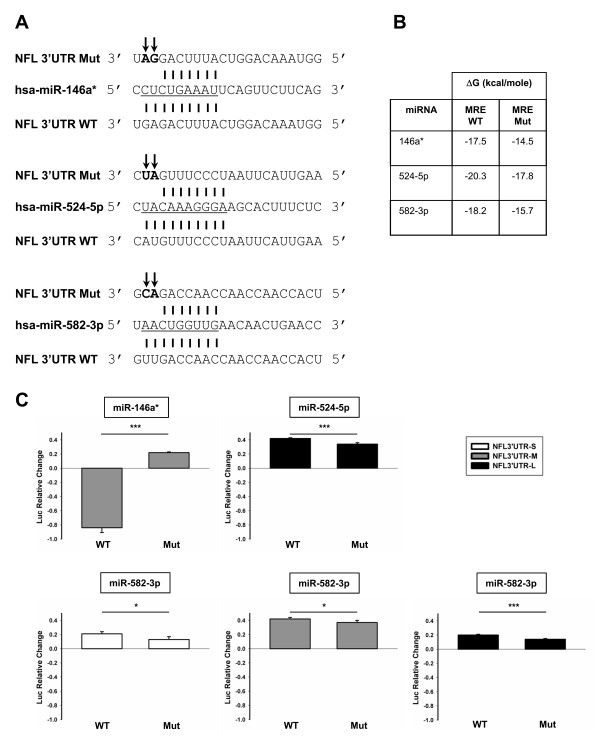
Mutations in the recognition elements of miR-146a*, miR-524-5p and miR-582-3p reveal a direct regulation of the miRNA on luciferase transcripts coupled to the NFL 3′UTR. (**A**) Mutants of MREs within the NFL 3′UTR. Only the first two nucleotides of the 3′ end of each MRE were mutated (arrows) preventing broad changes in the NFL 3′UTR secondary structure. (**B**) ΔGs of wild type (WT) and mutant (Mut) MREs within NFL 3′UTR. (**C**) Reporter gene assay performed with mutant NFL 3′UTRs. The results of firefly luciferase activity were normalized with Renilla reporter luciferase activity and the effect of each miRNA on the luciferase mRNA alone (pmirGLO vector control). Data are expressed as relative change and show up-regulation as positive values and down-regulation as negative values. Experiments were performed in triplicate. Result are shown as mean ± SEM (*t*-test: *** = p < 0.001; * = p < 0.05, relative to the pmirGLO vector control). All the NFL 3′UTR mutants showed a significant decrease in the effect of each miRNA compared with the wild type.

To study whether the regulatory effect of miR-146a*, miR-524-5p and miR-582-3p on the NFL 3′UTR was direct or indirect, we developed 3′UTR mutants of each MRE to test them with the reporter gene assay. We mutated only the first two nucleotides of the 3′ end of each miRNA recognition element thereby preventing broad changes in the NFL 3′UTR secondary structure that could interfere with the interpretation of our results (Figure 4A). Previously, it has been reported that mismatches at these positions significantly reduced the magnitude of target regulation [36]. In fact, two mutations within any of the three MREs analyzed (miR-146a*, miR-524-5p and miR-582-3p) further destabilized target recognition, yielding less favorable ΔGs (Figure 4B).

The reporter gene assay showed that all NFL 3′UTR MRE mutants exhibited a significant decrease in the effect of each miRNA compared with the wild type. Our data demonstrate that miR-146a*, miR-524-5p and miR-582-3p are able to directly regulate the NFL 3′UTR (Figure 4C).

All of our results demonstrate that a pool of three functionally relevant miRNAs capable of interacting with NFL mRNA 3′UTR, miR-146a*, miR-524-5p and miR-582-3p, are dysregulated in sALS, suggesting their participation in the regulation of NFL mRNA expression in mammalian cells. It is interesting to note that not all the miRNA found to be expressed at different levels in sALS served to solely down- or up-regulate the reporter linked to the NFL mRNA 3′UTR. These data strongly suggest that it will likely be a net effect of action of all miRNA involved in NFL expression, and not the action of one single miRNA, that is important for NFL regulation.

## Discussion

This study is the first comprehensive examination of the miRNA expression profile of SC tissue in ALS. We observed that the majority of dysregulated miRNAs in sALS (256) are down-regulated (246) and only a small group are up-regulated (10). This is the first report of a massive miRNA decrease compared with other neurodegenerative diseases [37-40]. Amongst those miRNAs that we observed to be differentially expressed in ventral lumbar SC in sALS compared to controls, we identified a panel of miRNAs with MREs in the human NFL mRNA 3′UTR that were able to regulate the expression of a luciferase reporter bearing this 3′UTR in mammalian cells.

We previously observed that RNA species could contribute to NFL mRNA stability in a series of *in vitro* experiments in which we incubated biotinylated human NFL mRNA with SC homogenates from control, sALS, mtSOD1-associated familial ALS (fALS), and non-mtSOD1 fALS and then assayed NFL mRNA levels [12]. Pretreatment of the SC homogenates with RNAse resulted in a significant increase in NFL mRNA stability, suggesting a role for RNA species that destabilizes NFL mRNA in ALS. Considering that miRNAs are key mRNA stability determinants, they presented a likely candidate to explain our previous results. To address this, we restricted our analysis to the lumbar SC given our longstanding interest in defining the mechanisms related to alterations in NFL mRNA stability associated with intraneuronal neurofilament aggregate formation in lumbar spinal motor neurons in ALS [41]. Moreover, we have also previously shown that alterations in NFL mRNA steady state levels observed in spinal motor neurons in ALS by *in situ* hybridization [15] could also be observed in ventral spinal cord tissue homogenates, but not in dorsal spinal cord homogenates or in tissues outside of the SC [42].

Of the group of miRNAs dysregulated in sALS SC that have MREs in the NFL 3′UTR, we found a significant increase in the expression of miR-146a*. This increase could be correlated with the decrease in NFL mRNA seen in ALS, given that this miRNA directly down-regulates the expression of a reporter linked to the NFL mRNA 3′UTR. Interestingly, miR-146a* strongly down-regulated the reporter coupled to NFL 3′ UTR-M but not when coupled to NFL 3′UTR-L. This result suggests that varying the length of the 3′UTR may alter the mRNA structure sufficiently to allow or disallow the interaction of miRNAs or other interactors such as RNA binding proteins, introducing another level of complexity to the regulation of NFL mRNA stability.

MiRNAs are thought to have predominantly a silencing effect on their targets. Although less common, miRNAs can also have either direct or indirect up-regulatory effects on mRNA expression. For example, miR-34 can interact with β-actin 3′UTR and positively regulating its expression [43], while miR-466 l up-regulates IL-10 mRNA expression in TLR-triggered macrophages by antagonizing RNA-binding protein tristetraprolin-mediated IL-10 mRNA degradation [44]. In our studies, we observed that two miRNAs whose expression levels were down-regulated in sALS (miR-524-5p and miR-582-3p) induced an increase of the reporter linked to the NFL mRNA 3′UTR. This reduced expression of miRNAs that can up-regulate NFL (such as miR-524-5p and miR582-3p) further supports our previous observations that NFL mRNA is selectively decreased in ALS.

Many recognition sites in the NFL mRNA 3′UTR for miRNAs dysregulated in sALS are conserved among different species. However, we have to consider that non-conserved sites can be as important, and can occur as frequently as conserved sites [45,46]. For example, almost half of all validated miRNA binding sites in the p21 mRNA 3′UTR were not evolutionarily conserved [47], indicating that exclusive attention to only conserved sites could bias against other important interactions. It is interesting that miR-146a*, miR-524-5p and miR-582-3p sites in the NFL mRNA 3′UTR are not conserved across species and all of them showed consistent results making them prime candidates for the down-regulation of NFL mRNA expression in the SC in ALS. This strongly suggests that these sites may be of importance, particularly in humans, for NFL regulation.

Of the three miRNAs that we showed could be involved in the selective decrease of NFL mRNA observed in ALS SC, miR-582-3p has been previously reported to have a possible role in the negative regulation of *Smad3*, one of the intracellular mediators of TGF-β. As TGF-β mediates apoptosis, down-regulation of miR-582-3p could additionally promote cell death [48]. This observation is in agreement with the IPA that showed that miRNAs differentially expressed in sALS SC are in general linked with cell death.

The complexity of the process is also highlighted by the observation that miRNAs can act singly or coordinately. We have experimentally confirmed that several miRNAs can regulate a reporter linked to NFL mRNA 3′UTR. This suggests that it may be the combination of expression and the cumulative effects of a group of miRNAs that are more important to overall regulation than the expression of any single miRNA. As well, the interaction of RNA binding proteins with NFL mRNA could alter the folded structure of the mRNA and as a result, alter miRNA access to their respective binding sites. Further defining the impact of sequestering RNA binding proteins in aggregates, which are commonly found in degenerating motor neurons in ALS, will be an imperative subject for future investigation. Taken together, this points to a complex regulatory system involving miRNA, which must be examined in the context of all regulatory elements (*trans* and *cis*) that may affect a single mRNA.

## Conclusions

This study provides the first evidence that the miRNA expression profile is broadly altered in the SC in sALS. Among miRNAs dysregulated in sALS, there is a group of (miR-146a*, 524-5p and 582-3p) that directly regulate the NFL mRNA 3′UTR, suggesting an involvement in the selective suppression of NFL mRNA in the spinal motor neurons in ALS.

## Methods

### Tissues

We selected 3 cases of non-neurological disease controls and 5 cases of sALS (Table 6). All ALS cases were both clinically and neuropathologically confirmed using the El Escorial Criteria (World Federation of Neurology Research Group on Neuromuscular Disease, 1994). Written consent for tissue donation at autopsy was obtained from either the patient (ante-mortem) or the spouse (post-mortem) in accordance with the London Health Sciences Centre ethics policies. All cases were genotyped and confirmed to have no known mutations in SOD1, *TARDBP*, FUS/TLS or expanded repeats on C9ORF72 by Dr. Rosa Rademakers (Mayo Clinic, Jacksonville, Florida). No cases were associated with cognitive impairment or frontotemporal degeneration.

**Table 6:** Patient demographics for SC tissues included in this study

**Case**	**Gender**	**Age at symtom onset (yrs)**	**Age at death (yrs)**	**Symptom onset**	**Cause of death**
Control	F	-	53	-	Cardiac transplant, pneumonia, rejection
Control	M	-	68	-	Cancer
Control	F	-	62	-	Ischemic heart disease, myocardial infarct
ALS	F	47	49	Bulbar	Respiratory failure
ALS	F	Unknown	41	Bulbar	Respiratory failure
ALS	M	55	61	Unknown	Pneumonia
ALS	M	40	44	Spasticity	Unknown
ALS	M	64	67	Upper limb, weakness	Respiratory failure

-: Not applicable.

### MiRNA extraction

MiRNAs were extracted using the mirVana™ miRNA isolation kit (Life Technologies Inc., Ambion, Burlington, ON, Canada) according to the manufacturer’s instructions. Briefly, archival ventral lumbar SC tissue stored at −80°C was placed in 10 volumes of lysis/binding buffer on ice and homogenized with a Polytron until all visible clumps were dispersed. After organic extraction with acid-phenol:chloroform, miRNA isolation was performed by precipitating with ethanol and purifying over two consecutive glass-fiber filters. This procedure yielded a size-selected RNA fraction consisting of RNA species of less than 200 bases. Yield and purity of the miRNA enriched fraction was measured by UV absorbance at OD260/280. Quality of RNA samples was confirmed by BioAnalyzer (Agilent).

### MiRNA real-time qRT-PCR

The stem-loop RT-PCR based TaqMan Human MicroRNA Arrays (Life Technologies Inc., Applied Biosystems, Burlington, ON, Canada) were used representing 664 mature miRNA in the Sanger miRBase v12 in a two-card set of arrays (Array A and B). Array A contained 3 positive controls and 1 negative control while Array B contained 6 positive controls and 1 negative control. RT-PCR reactions were performed according to the manufacturer’s instructions. Briefly, 300 ng of enriched miRNA (extracted as described above) was reverse transcribed using Megaplex RT Primers and the TaqMan miRNA reverse transcription kit (Life Technologies Inc., Applied Biosystems). For pre-amplification of miRNA cDNA, the RT product was pre-amplified using Megaplex PreAmp Primers and TaqMan PreAmp Master Mix as per manufacturer’s protocols (Life Technologies Inc., Applied Biosystems). The pre-amplified cDNA was diluted with 0.1 × TE (pH 8.0) to 100 μl and then 10 μl of the cDNA was used in each plate for RT-qPCR reaction.

Quantitative real-time PCR (RT-qPCR) was performed using the Applied Biosystems 7900HT system and TaqMan Universal Master Mix (Life Technologies Inc., Applied Biosystems). Cycle threshold (Ct) values were calculated using the SDS software v.2.3. Ct values > 40 were considered to be below the detection level of the assay, therefore, only the miRNAs with Ct ≤ 40 were included in the analyses.

For the data analysis the Ct value of the endogenous mammalian U6 control was subtracted from the corresponding Ct value for the target gene resulting in the normalized ΔCt value. The Student’s *t* test was used to determine significant differences among ΔCt values of control and sALS groups. The differential expression (the difference between the average of normalized ΔCt value of the target sample (sALS) and the average of normalized ΔCt value for the corresponding calibrator sample (control); ΔΔCT), was used to calculate the expression fold value as follows:

$${\mathrm{Log}}_{10}\mathrm{RQ}={\mathrm{Log}}_{10}2^{-}{}^{\Delta \left(\Delta \mathrm{CT}\right)}$$

Where Log_10_RQ correlates directly with up- (positive value) and down-regulation (negative value).

For control cases, the results were deemed negative if all cases were negative for expression. For sALS cases, agreement amongst 4 or more cases was required to consider the result either positive or negative.

### Analysis of miRNA target genes

We used Ingenuity Pathway Analysis (IPA, 368 Ingenuity® Systems, Redwood City, CA) to analyze dysregulated miRNAs in the SC in ALS. IPA allows us to identify over-represented networks and biological functions of miRNA target genes. The networks are scored with a numerical value and a score ≥ 2 was considered significant. The score is based on the number of Network Eligible molecules in the network, the size of the network, the Network Eligible molecules in the given dataset and the number of molecules in the IPA dataset that could potentially be included in the network. The p-value associated with a biological process is calculated with the Fisher’s exact test, considering the number of functions/pathways/lists of eligible molecules that participate in the annotation, the total number of knowledge base molecules known to be associated with that function, the total number of functions/pathways/lists eligible molecules and the total number of genes in the Reference Set (IPA tutorial).

### MiRNA target prediction

To determine if miRNAs differentially expressed in sALS have miRNA recognition elements within the NFL mRNA 3′UTR we used two miRNA target prediction sites: microRNA.org (http://www.microrna.org/microrna/getGeneForm.do) [49] and TargetScan (http://www.targetscan.org/) [45].

### Plasmid construction

Three reporter plasmids were constructed by inserting human NFL 3′UTR of different lengths between NheI and SalI sites downstream of the firefly luciferase gene in the pmirGLO vector (Promega, Madison, WI, USA; pmirGLO-NFL 3′UTR). NFL 3′UTR-Short (−S) encoded the shortest predicted isoform homologous to the murine NFL mRNA (GenBank NM_010910) consisting of 1–286 b of the 3′UTR. NFL 3′UTR-Medium (−M) included bases 1–1380 (GenBank BC039237), and NFL 3′UTR-Long (−L) comprised bases 1–1838 (GenBank NM_006158). Mutations in two nucleotides of each MRE of the NFL 3′UTR were introduced using QuikChange Lightning Site-Directed Mutagenesis kit (Agilent Technologies Canada Inc., Mississauga, ON, Canada) according to the manufacturer’s instructions. Mutagenesis was designed carefully preventing broad changes in the NFL 3′UTR secondary structure using the mfold web server (http://mfold.rit.albany.edu/?q=mfold/RNA-Folding-Form) [50]. ΔG values for the interaction among each miRNA and MREs were calculated using RNAhybrid server [51].

### Cell culture and transfection

To perform functional analysis, HEK293T cells were maintained in Dulbecco’s modified Eagle’s medium (DMEM) containing 10% FBS and plated 24 h prior to transfection in 96-well plates at 9 × 10^3^ cells/well. Cells were co-transfected with 100 nM of pre-miRNAs (Life Technologies Inc., Ambion) and 3.47 fmol of pmirGLO-NFL 3′UTR (−S, -M or -L) using Lipofectamine 2000 reagent (Life Technologies Inc., Invitrogen, Burlington, ON, Canada), according to the manufacturer’s instructions.

### Luciferase reporter assay

Luciferase activity was measured 24 h after transfection using the Dual-Glo Luciferase Assay System (Promega) in a Luminometer (Promega, Turner Biosystems, Madison, WI, USA). Firefly luciferase activity was normalized to Renilla luciferase activity to adjust for variations in transfection efficiency among experiments. Data were also normalized to the impact of each miRNA on the luciferase mRNA without NFL 3′UTR to obtain the specific effect on the NFL mRNA 3′UTR. All experiments were performed in triplicate. Quantitative data of the reporter gene assay are presented as mean ± SEM. The Student’s *t-*test was used to determine significant differences between two groups.

### Relative quantitative RT-PCR

24 h after transfection total RNA was isolated using TRIzol reagent (Life Technologies Inc., Ambion) according to the manufacturer’s instructions. After the reverse transcription, relative quantitative PCR was carried out co-amplifying Firefly and Renilla luciferases encoded in the same pmirGLO vector using the following primers: *fLuc:* forward 5′ CAA GAC TAT AAG ATT CAA TCT GCC CTG CTG 3′ and reverse 5′ GAT GTT GGG GTG TTG CAG CAG GAT 3′; *rLuc:* forward 5′ GAG CAA CGC AAA CGC ATG ATC ACT G 3′ and reverse 5′ TTC AGC AGC TCG AAC CAA GCG GT 3′.

The intensity of the bands from the agarose gel was quantified by densitometry. Firefly luciferase densitometry was normalized to Renilla luciferase densitometry to adjust for variations in transfection efficiency among experiments. Data were also normalized to the impact of each miRNA on the luciferase mRNA without NFL 3′UTR and to the effect of miR-let-7a (miRNA control) on the luciferase mRNA to obtain the specific effect on the NFL mRNA 3′UTR. All experiments were performed in triplicate. The Student’s *t-*test was used to determine significant differences between two groups.

## Abbreviations

ALS: Amyotrophic lateral sclerosis; fALS: Familial ALS; sALS: Sporadic ALS; C9ORF72: Chromosome 9 open reading frame 72; FUS/TLS: RNA processing protein fused in sarcoma/translocated in liposarcoma; IL-10: Interleukin 10; IPA: Ingenuity pathway analysis; miRNA: microRNA; MRE: miRNA recognition element; NF: Neurofilament; NFL: Low molecular weight neurofilament; NFL 3′ UTR-S: Short NFL 3^′^ UTR; NFL 3′ UTR-M: Medium NFL 3^′^ UTR; NFL 3′ UTR-L: Long NFL 3^′^ UTR; P-bodies: Processing bodies; RGNEF: Rho guanine nucleotide exchange factor; SC: Spinal cord; SOD1: Cu/Zn superoxide dismutase 1; mtSOD1: Mutant SOD1; TARDBP: Gene encoding TAR DNA binding protein 43 kDa; TDP-43: TAR DNA binding protein 43 kDa; TGF-β: Transforming growth factor-β; TLR: Toll-like receptor; 3′ UTR: 3^′^ untranslated region.

## Competing interests

The authors declare that they have no competing interests.

## Authors’ contributions

DCM, CAD and ZH performed the experiments and analyzed the data included in the manuscript. DCM, KV and MJS conceived the experiments and wrote the manuscript. All authors read and approved the final manuscript.

## Supplementary Material

Additional file 1 miRNA expression profile in ALS. Click here for file
