# Supplementary material for: Altered microRNA expression profile in amyotrophic lateral sclerosis: a role in the regulation of NFL mRNA levels
Source: Mol Brain. 2013 May 24;6:26. doi: 10.1186/1756-6606-6-26 (PMC3668997; doi:10.1186/1756-6606-6-26)
Supplement: Additional file 1 — miRNA expression profile in ALS. [file 1756-6606-6-26-S1.doc]

**Supplemental Table 1**

MicroRNAs expressed in both sALS and controls but down-regulated in sALS.

| **miRNA** | **P-value** | **Log_10_RQ** |
| --- | --- | --- |
| 1 | 0.010 | -0.987128 |
| 9 | 0.020 | -0.818178 |
| 10a | 0.026 | -0.814665 |
| 10b | 0.025 | -1.027079 |
| 15a | 0.028 | -0.602830 |
| 15b | 0.032 | -0.999565 |
| 17 | 0.027 | -0.531981 |
| 18a | 0.037 | -1.163879 |
| 18b | 0.030 | -1.176139 |
| 19a | 0.024 | -0.691166 |
| 19b | 0.037 | -0.729382 |
| 20a | 0.030 | -0.522824 |
| 20b | 0.034 | -0.707627 |
| 22 | 0.015 | -0.827208 |
| 23a | 0.017 | -1.026973 |
| 23b | 0.020 | -1.147540 |
| 24 | 0.044 | -0.613151 |
| 25 | 0.023 | -0.813261 |
| 27a | 0.040 | -0.889380 |
| 27b | 0.028 | -1.086063 |
| 28-3p | 0.042 | -0.673076 |
| 28-5p | 0.035 | -0.849152 |
| 29a | 0.023 | -0.745617 |
| 29b | 0.008 | -1.153710 |
| 29c | 0.035 | -0.582517 |
| 30b | 0.017 | -0.757786 |
| 30c | 0.018 | -0.600730 |
| 31 | 0.046 | -0.594426 |
| 34a | 0.015 | -1.127136 |
| 34c-5p | 0.048 | -0.738334 |
| 92a | 0.034 | -1.051463 |
| 93 | 0.027 | -0.875791 |
| 98 | 0.039 | -0.842555 |
| 99a | 0.022 | -0.806438 |
| 99b | 0.018 | -1.375729 |
| 99b* | 0.016 | -0.453379 |
| 100 | 0.026 | -0.765490 |
| 101 | 0.033 | -0.657273 |
| 103 | 0.012 | -0.955134 |
| 105 | 0.011 | -0.915997 |
| 106a | 0.042 | -0.510013 |
| 106b | 0.015 | -0.745209 |
| 107 | 0.015 | -1.154066 |
| 122 | 0.011 | -2.299955 |
| 124 | 0.023 | 0.825869 |
| 125a-3p | 0.011 | -0.720821 |
| 125a-5p | 0.008 | -0.571466 |
| 125b | 0.008 | -0.999562 |
| 127-3p | 0.006 | -0.921678 |
| 127-5p | 0.017 | -1.428855 |
| 128 | 0.029 | -1.093481 |
| 129-3p | 0.007 | -1.240759 |
| 129-5p | 0.007 | -0.841542 |
| 130a | 0.049 | -0.773127 |
| 130b | 0.008 | -0.915665 |
| 132 | 0.045 | -0.510850 |
| 133a | 0.010 | -0.673076 |
| 133b | 0.008 | -1.048894 |
| 135a | 0.035 | -0.495129 |
| 135b | 0.036 | -0.601321 |
| 137 | 0.014 | -0.922444 |
| 138-1* | 0.041 | -0.530998 |
| 139-3p | 0.007 | -1.110191 |
| 139-5p | 0.031 | -0.561359 |
| 140-5p | 0.047 | -0.740085 |
| 141 | 0.025 | -0.559016 |
| 142-3p | 0.020 | -0.818129 |
| 142-5p | 0.024 | -0.854246 |
| 146b-3p | 0.032 | -0.846451 |
| 147 | 0.006 | -2.419374 |
| 148b | 0.021 | -0.729676 |
| 149 | 0.005 | -0.834845 |
| 150 | 0.039 | -0.651067 |
| 152 | 0.030 | -0.599043 |
| 153 | 0.012 | -1.057926 |
| 154 | 0.011 | -0.933762 |
| 181a | 0.018 | -0.658103 |
| 181c | 0.014 | -0.692949 |
| 184 | 0.026 | -0.584630 |
| 185 | 0.008 | -0.850913 |
| 188-3p | 0.026 | -2.056480 |
| 190 | 0.003 | -0.726530 |
| 192 | 0.026 | -0.517389 |
| 193a-3p | 0.018 | -0.617249 |
| 193a-5p | 0.017 | -0.878426 |
| 193b | 0.006 | -0.551935 |
| 194 | 0.027 | -0.823008 |
| 197 | 0.029 | -0.698711 |
| 198 | 0.031 | -0.778428 |
| 200a | 0.016 | -0.704926 |
| 200b | 0.025 | -0.620496 |
| 202 | 0.033 | -1.876294 |
| 204 | 0.020 | -0.736086 |
| 210 | 0.029 | -0.909838 |
| 211 | 0.037 | -0.935401 |
| 212 | 0.035 | -0.413280 |
| 215 | 0.031 | -0.630715 |
| 216a | 0.011 | -0.967741 |
| 216b | 0.005 | -0.803707 |
| 218 | 0.019 | -0.992982 |
| 218-2* | 0.043 | -1.091421 |
| 219-5p | 0.010 | -1.192686 |
| 219-2-3p | 0.014 | -1.078703 |
| 221 | 0.027 | -0.797339 |
| 223 | 0.035 | -0.656259 |
| 296-3p | 0.021 | -0.913668 |
| 296-5p | 0.033 | -0.986206 |
| 299-3p | 0.024 | -1.409041 |
| 299-5p | 0.036 | -1.343748 |
| 301a | 0.007 | -0.849642 |
| 301b | 0.012 | -0.808218 |
| 302a | 0.019 | -0.674257 |
| 302c | 0.041 | -0.662274 |
| 320 | 0.030 | -0.415364 |
| 323-3p | 0.010 | -0.905325 |
| 324-3p | 0.026 | -0.665923 |
| 324-5p | 0.023 | -1.262101 |
| 326 | 0.007 | -1.290653 |
| 328 | 0.011 | -1.053664 |
| 329 | 0.009 | -1.229222 |
| 330-3p | 0.005 | -1.025828 |
| 330-5p | 0.008 | -0.955733 |
| 331-3p | 0.015 | -0.692112 |
| 331-5p | 0.007 | -0.540036 |
| 335 | 0.048 | -0.588378 |
| 337-5p | 0.028 | -1.052604 |
| 338-3p | 0.041 | -0.644606 |
| 339-3p | 0.039 | -0.371057 |
| 339-5p | 0.014 | -0.745130 |
| 340 | 0.026 | -0.745831 |
| 342-5p | 0.027 | -0.717941 |
| 345 | 0.040 | -0.603088 |
| 346 | 0.010 | -1.118977 |
| 361-5p | 0.005 | -0.815424 |
| 362-3p | 0.027 | -0.712057 |
| 362-5p | 0.048 | -0.738334 |
| 363 | 0.026 | -0.866100 |
| 365 | 0.030 | -1.165933 |
| 367 | 0.021 | -0.700001 |
| 367* | 0.002 | -6.168528 |
| 369-3p | 0.041 | -0.632008 |
| 369-5p | 0.023 | -1.311960 |
| 370 | 0.013 | -0.928535 |
| 376a | 0.030 | -0.507430 |
| 377 | 0.011 | -1.235061 |
| 379 | 0.009 | -0.946753 |
| 380 | 0.011 | -1.055358 |
| 381 | 0.008 | -1.120339 |
| 382 | 0.025 | -0.991910 |
| 383 | 0.017 | -0.883940 |
| 409-5p | 0.010 | -1.221568 |
| 422a | 0.049 | -0.855792 |
| 423-5p | 0.019 | -1.190908 |
| 424 | 0.020 | -0.771598 |
| 425 | 0.023 | -0.822209 |
| 425* | 0.048 | -0.341659 |
| 433 | 0.020 | -1.030514 |
| 448 | 0.012 | -0.917048 |
| 450a | 0.036 | -0.877224 |
| 450b-5p | 0.037 | -0.490000 |
| 455-3p | 0.033 | -0.887322 |
| 455-5p | 0.047 | -0.821813 |
| 485-3p | 0.010 | -0.940370 |
| 485-5p | 0.008 | -1.153338 |
| 487a | 0.017 | -1.020598 |
| 487b | 0.038 | -0.582667 |
| 490-3p | 0.008 | -1.344799 |
| 491-5p | 0.009 | -0.675257 |
| 494 | 0.020 | -0.918529 |
| 495 | 0.015 | -0.982619 |
| 499-5p | 0.011 | -0.829105 |
| 500 | 0.014 | -0.835140 |
| 501-5p | 0.022 | -0.911408 |
| 502-3p | 0.024 | -0.790548 |
| 502-5p | 0.025 | -0.544805 |
| 503 | 0.041 | -0.650923 |
| 504 | 0.011 | -0.995965 |
| 505 | 0.024 | -1.042783 |
| 511 | 0.047 | -0.583013 |
| 512-3p | 0.048 | -0.489725 |
| 515-5p | 0.027 | -1.280364 |
| 516b | 0.044 | -1.043670 |
| 517a | 0.042 | -0.692823 |
| 519a | 0.032 | -0.745382 |
| 519d | 0.038 | -0.619331 |
| 520a-3p | 0.035 | -0.951238 |
| 520a-5p | 0.012 | -2.414146 |
| 520f | 0.031 | -1.647647 |
| 532-3p | 0.024 | -0.815834 |
| 532-5p | 0.028 | -0.842934 |
| 539 | 0.016 | -0.760941 |
| 541 | 0.040 | -0.669030 |
| 542-3p | 0.022 | -0.811985 |
| 544 | 0.039 | -0.842850 |
| 545 | 0.020 | -0.744681 |
| 548c-5p | 0.018 | -1.075390 |
| 548d-5p | 0.019 | -0.924270 |
| 550* | 0.012 | -0.628206 |
| 551b | 0.030 | -0.999976 |
| 556-5p | 0.034 | -2.063602 |
| 570 | 0.030 | -0.595193 |
| 574-3p | 0.039 | -0.441318 |
| 576-3p | 0.023 | -0.722488 |
| 576-5p | 0.018 | -1.075809 |
| 578 | 0.008 | -1.108681 |
| 579 | 0.015 | -0.980217 |
| 582-3p | 0.005 | -0.817983 |
| 582-5p | 0.039 | -1.574908 |
| 589 | 0.028 | -0.844236 |
| 597 | 0.011 | -1.054688 |
| 598 | 0.021 | -0.794276 |
| 604 | 0.011 | -0.742681 |
| 618 | 0.037 | -1.191667 |
| 625 | 0.017 | -0.876517 |
| 625* | 0.038 | -0.534958 |
| 627 | 0.034 | -0.719987 |
| 629 | 0.045 | -1.120159 |
| 642 | 0.003 | -1.443308 |
| 651 | 0.018 | -0.788494 |
| 652 | 0.029 | -0.718745 |
| 654-3p | 0.008 | -1.144359 |
| 654-5p | 0.016 | -0.950953 |
| 655 | 0.015 | -0.854805 |
| 660 | 0.036 | -0.783868 |
| 671-3p | 0.004 | -0.854086 |
| 708 | 0.027 | -0.783603 |
| 744 | 0.012 | -0.879403 |
| 758 | 0.016 | -1.036352 |
| 760 | 0.048 | -0.431389 |
| 873 | 0.015 | -0.947619 |
| 874 | 0.017 | -1.168408 |
| 885-5p | 0.010 | -0.933480 |
| 887 | 0.044 | -0.576273 |
| 889 | 0.009 | -0.903116 |
| let-7a | 0.020 | -0.687876 |
| let-7b | 0.020 | -0.848993 |
| let-7c | 0.043 | -0.707562 |
| let-7d | 0.032 | -0.768614 |
| let-7e | 0.030 | -0.738057 |

**Supplemental Table 2**

MicroRNAs that are not expressed in either control or sALS spinal cord lysates.

| 325 | 588 |
| --- | --- |
| 384 | 603 |
| 517* | 607 |
| 518c* | 608 |
| 548c-3p | 888* |
